# Epigenetic restoration and activation of ERβ: an inspiring approach for treatment of triple-negative breast cancer

**DOI:** 10.1007/s12032-022-01765-1

**Published:** 2022-07-18

**Authors:** Ahmad Salahuddin, Heba Ghanem, Gamal A. Omran, Maged Wasfy Helmy

**Affiliations:** 1 Department of Biochemistry, Faculty of Pharmacy, Damanhour University, Damanhour, 22511 Egypt; 2 Department of Pharmacology & Toxicology, Faculty of Pharmacy, Damanhour University, Damanhour, 22511 Egypt

**Keywords:** TNBC, DNMTI, Decitabine, HDACI, Vorinostat, ERβ agonist, DPN, MDA-MB-231

## Abstract

**Background:**

Triple-negative breast cancer (TNBC) is one of the most aggressive subtypes of breast cancer. TNBC lacks targeted therapy receptors, rendering endocrine and HER2-targeted therapies ineffective. TNBC is typically treated with cytotoxic chemotherapy followed by surgery. Targeting epigenetic modifications could potentially be a new effective TNBC target therapy. The aim of this study is to examine the effects of epigenetic drugs, decitabine as DNA methyltransferase inhibitor (DNMTI) and vorinostat as histone deacetylase inhibitor (HDACI), and the ERβ agonist DPN on ERα and ERβ re-expressions in the MDA-MB-231 cells as a model of TNBC.

**Methods:**

Using MTT assay, the IC_50_ of decitabine, vorinostat, and DPN on MDA-MB-231 cells were determined. The effects of all drugs alone or in combinations on MDA-MB-231 cells were evaluated. qRT-PCR was used to determine ERα & ERβ gene expression. Caspase-3 activity and the protein expression levels of VEGF, Cyclin D1, and IGF-1 were assessed.

**Results:**

Both ERα and ERβ mRNA were re-expressed in different high levels in all treated groups, especially in the triple therapy group compared with control. Significantly, the triple drugs therapy showed the lowest levels of VEGF, Cyclin D1, and IGF-1 and the highest level of Caspase-3 activity, indicating a possible antitumor effect of ERβ activation through decreasing proliferation and angiogenesis and increasing apoptosis in MDA-MB-231 cells.

**Conclusions:**

The antiproliferative effect of ERβ could be retained when co-expressed with Erα using a powerful epigenetic combination of Decitabine and vorinostat with DPN.

## Introduction

Worldwide, breast cancer (BC) is the most diagnosed cancer and the leading cause of cancer death in women, with 24.5% incidence and 15.5% mortality according to global cancer statistics 2020 [1]. Triple-negative breast cancer (TNBC) is one of the most aggressive subtypes of BC accounting for 15–20% of all BC cases but is responsible for over 50% of BC mortality. TNBC has limited treatment options due to the lack of expression of three receptors: the estrogen receptor alpha (ERα), progesterone receptor (PR), and the human epidermal growth factor receptor 2 (HER2) amplification [2]. Therefore, current hormonal or HER2-targeted therapies are not viable for TNBCs; cytotoxic chemotherapy is still the only standard treatment option available despite harsh side effects [3, 4]. Although TNBC patients have a better clinical response to chemotherapy, they have a worse prognosis than other BC subtypes. [5]. Therefore, there is an urgent need to identify better treatment options that are less toxic and are more targeted to TNBC patients.

Despite the lack of ERα in TNBCs, the discovery of estrogen receptor beta (ERβ) expression in some TNBC subtypes made it a possible logical therapeutic target [6, 7]. Unlike the tumorigenesis effect of ERα, ERβ has been suggested to act as a tumor suppressor in breast tissue because its expression declines during carcinogenesis, its knockdown increases the proliferation of mammary epithelial and BC cells. In contrast, its overexpression inhibited tumor cells proliferation, acting as a brake [8, 9]. In general, ERβ activity is considered antagonistic to that of ERα when both receptors co-expressed together in a cell [10]; thus, activation of ERβ by specific agonists is suggested to be a feasible treatment option for BC, including TNBC [11]. One of the first synthetic ERβ selective agonists reported to have a high affinity for ERβ is DPN (2,3-Bis (4-hydroxyphenyl) propionitrile, diaryl propionitrile), which was used to examine the role of ERβ in different TNBC studies [5].

Epigenetic modifications, such as DNA methylation and histones acetylation, are heritable epigenetic processes that regulate gene expression in normal mammalian development [12]. TNBCs show extensive promoter hypermethylation of critical genes such as tumor suppressors and ERs compared with other BC subtypes; thus, targeting epigenetic regulators showed promising benefits in a series of TNBC cells [13].

It is well-known that the epigenetic silencing of the ERα gene in ER-negative human BCs involves interactions between DNA methyltransferases (DNMTs) and histone deacetylases (HDACs), which associated with DNA hypermethylation and histone hypoacetylation to maintain a stable repressive chromatin complex in the silenced ER promoter [12]. In harmony, studies demonstrated that ERβ expression is regulated by DNA methylation and histone acetylation. Hypermethylation of ESR2 promoter was associated with a marked decrease of ERβ mRNA expression in BCs, while inhibition of DNMTs reactivated ERβ expression. In both ERα -positive luminal and ERα -negative basal-like BC cells, HDAC inhibitors (HDACIs) increased ERβ expression [14]. Therefore, several studies supported using a combination of DNMT inhibitors (DNMTIs) and HDACIs for ERs re-expression in TNBCs [12].

Decitabine (5-aza-2′deoxycytidine) is a DNMTI that is approved by the US-FDA for treating hematological malignancies. Decitabine suppresses all 3 DNMTs: DNMT1, DNMT3A, and DNMT3B causing partial demethylation of the ER CpG island and restores expression of functional ER in ER-negative human BC cells [13]. Vorinostat (Suberanilohydroxamic acid, SAHA) HDACI, is the first in its class to be approved by the US-FDA to treat cutaneous T-cell lymphoma. It inhibits class I and II HDACs, including HDAC1, HDAC2, HDAC3, and HDAC6, at low micromolar concentrations [12].

There is mounting evidence that a combination of HDACI, such as vorinostat, with DNMTI, such as decitabine, can restore ERα expression and sensitize ER-negative BCs like MDA-MB-231 cells to hormone therapy or chemotherapy [12]. Therefore, reactivating both of ERα and ERβ expressions in MDA-MB-231 TNBC cells that are a well-characterized model that does not endogenously express any form of the ERs [6] could restore the antiproliferative effect of ERβ in the presence of ERα using a selective ERβ agonist such as DPN.

In the present study, we examine the antitumor effect of DPN (ERβ agonist) after re-expression of ERα and ERβ using the powerful epigenetic combination of Decitabine (DNMTI) and vorinostat (HDACI) for treatment of MDA-MB-231 cells (TNBC cell line).

## Materials and methods

### Chemicals

Decitabine and Vorinostat (code: s1200 and s1047, respectively) were purchased from SelleckChem, USA. DPN (code: 1494) was purchased from Tocris Bioscience, (UK). All other chemicals and materials were commercially available and of standard quality.

### Experimental cell lines

The MDA-MB-231 cell line was supplied from the American Type Culture Collection (ATTC, Manassas, VA, USA). According to method described previously [15], Cells were cultured in DMEM supplemented with 1% penicillin/streptomycin and 10% FBS and incubated at 37 °C in the presence of 5% CO2 and 95% humidified air. Cells were harvested at 80% confluence using a 2.5% (w/v) trypsin solution and subculture into T-75 flasks or 96-well plates, depending on the experiment.

### Cell viability assay

According to the method described previously [15], The MTT assay was used to determine the effects of Decitabine, Vorinostat, or DPN on cell viability. MDA-MB-231 cells were seeded in a 96-well plate at a density of 4000 cells per well, with each well containing 100 µl DMEM medium supplemented with 10% FBS and incubated overnight at 37 °C in 5% CO_2_, 95% air until 70–80% confluence.

The Old media was aspirated and then 200 μl of DMEM containing different drug concentrations was added to all wells except control wells and incubated for another 72 h.

- Decitabine concentrations (0.5, 1, 2, 4, 8 and 16 μM) [16].
- Vorinostat concentrations (0.0187, 0.0375, 0.075, 0.15, 0.3 and 0.6 μM) [17].
- DPN concentrations (0.005, 0.01, 0.02, 0.04, 0.08 and 0.16 μM) [5].

The media were then aspirated, and cells were incubated in the dark for four hours in the dark with 20 μl MTT working solution (5 mg/ml in DMEM). After removing the supernatant, the resulting purple formazan crystals were dissolved in 150 μl of DMSO over a 15 min period of agitation. Absorbance was recorded at 590 nm using a microplate reader. Each experiment was repeated at least three times independently in triplicate. As mentioned in previous study [18], the cells’ viability was expressed as a percentage relative to control. Median inhibitory concentration (IC_50_) values were determined using CompuSyn software (CompuSyn, Inc., version 1).

### Experimental design and treatment of MDA-MB-231 cells with drugs

At first, equal numbers of cells (2 × 10^5^ cells/flask) [15] were cultured in 24 identical T-25 culture flasks and incubated at 37 °C in 5% CO_2_. After 48 h of incubation, cell viability and confluency of the flasks were checked to be 70–80%.

The 24T-25 seeded flasks were divided into eight groups each group contained three flasks, treated as follows:

- Group I (*n* = 3): control group treated with complete media only as a vehicle.
- Group II (*n* = 3): treated with Decitabine (4 μM)
- Group III (*n* = 3): treated with Vorinostat (0.26 μM)
- Group IV (*n* = 3): treated with DPN (0.093 μM)
- Group V (*n* = 3): treated with Decitabine (4 μM) and Vorinostat (0.26 μM)
- Group VI (*n* = 3): treated with DPN (0.093 μM) and Vorinostat (0.26 μM)
- Group VII (*n* = 3): treated with DPN (0.093 μM) and Decitabine (4 μM)
- Group VIII (*n* = 3): treated with DPN (0.093 μM), Vorinostat (0.26 μM) and Decitabine (4 μM)

All treatments were applied at 70–80% confluence, and the cells were incubated in a CO_2_ incubator for 72 h; then, the cells were harvested and portioned into aliquots. The total protein content of each aliquot was quantified via the method reported by Bradford MM, 1976 [19]. Finally, the aliquots were kept at − 80 °C for further investigations.

### Preparation of cell lysates

Cell lysates were prepared using RIPA lysis and extraction buffer, purchased from Thermo Scientific, USA (Catalog Number: 89900). As directed by the manufacturer, 1 ml of cold RIPA buffer was added to 40 mg of wet cell pellets, which were then kept on ice and gently shaken for 15 min and then centrifuged at 14,000×*g* for 15 min to pellet the cell debris. After that, the supernatants were transferred to new tubes and stored at 20 °C for subsequent analysis.

### Biomarker analysis using ELISA technique

Vascular endothelial growth factor (VEGF), insulin-like growth factor 1 (IGF-1), and cyclin D1 were evaluated in the cell lysates from different treatment groups using the ELISA technique using Human VEGF ELISA kit (Cusabio Biotech Co., LTD, China, code: CSB-E11718h), and Human IGF-1 ELISA kit (Abnova, USA, code: KA0349), and Human Cyclin-D1 ELISA kit (Eiaab Science Inc, Wuhan, China, code: E0585h).

The manufacturer's protocol was followed in all measurements. Each parameter was assayed in triplicate and was expressed relative to the total protein content in the same sample.

### Caspase‑3 activity assay

Caspase-3 activity was measured using a colorimetric kit (Caspase-3 Assay Kit, Colorimetric (Sigma-Aldrich, USA, code: CASP-3-C), according to the manufacturer's procedure.

### Quantitative real-time polymerase chain reaction (qRT‑PCR)

According to the manufacturer’s protocols, A total RNA extraction kit, easy-RED™ Total RNA Extraction Kit (iNtRON Biotechnology, S.Korea, Catalog Number: 17063), was used to extract total RNA, then The RNA was reverse transcribed using a TOPscript™ cDNA Synthesis kit (Enzynomics, S.Korea, Catalog Number: EZ005S). Using qRT-PCR (DTlite Real-Time PCR system), ERα and ERβ gene expression was determined by a TOPreal™ qPCR 2X PreMIX (SYBR Green with low ROX) (Enzynomics, S.Korea, Catalog Number: RT500S) using a housekeeping gene, glyceraldehyde-3-phosphate dehydrogenase (GAPDH). The primer pair sequences are shown in Table 1. The assessment of each specimen was carried out in triplicate, and the fold changes in ERα and ERβ gene expression were calculated as described by Livak et al. [20].

**Table 1:** Primers sequences used for qRT-PCR

Genes	Sequences
Erβ	Forward (F): 5′-ACT TGC TGA ACG CCG TGA CC-3′ Reverse (R): 5′-CCA TCG TTG CTT CAG GCA A-3′
Erα	Forward (F): 5′-TGC CCT ACT ACC TGG AGA ACG-3′ Reverse (R): 5′-GTC CTT CTC TTC CAG AGA C-3′
GAPDH	Forward (F): 5′-ACC ACA GTC CAT GCC ATC AC-3′ Reverse (R): 5′-TCC ACC ACC CTG TTC CTG TA-3′

### Statistical analysis

The data were expressed as the mean ± standard error of the mean (SEM). Multiple comparisons were analyzed using a one-way analysis of variance (ANOVA) followed by a post hoc Tukey’s multiple comparison test., and the differences were considered significant at *p* < 0.05. GraphPad Prism® software package version 8.0.2 (GraphPad Software Inc., CA, USA) was used for all statistical analyses and data presentation.

## Results

### IC_50_ values of decitabine, vorinostat, and DPN in MDA-MB-231 cell line

**Fig. 1:**
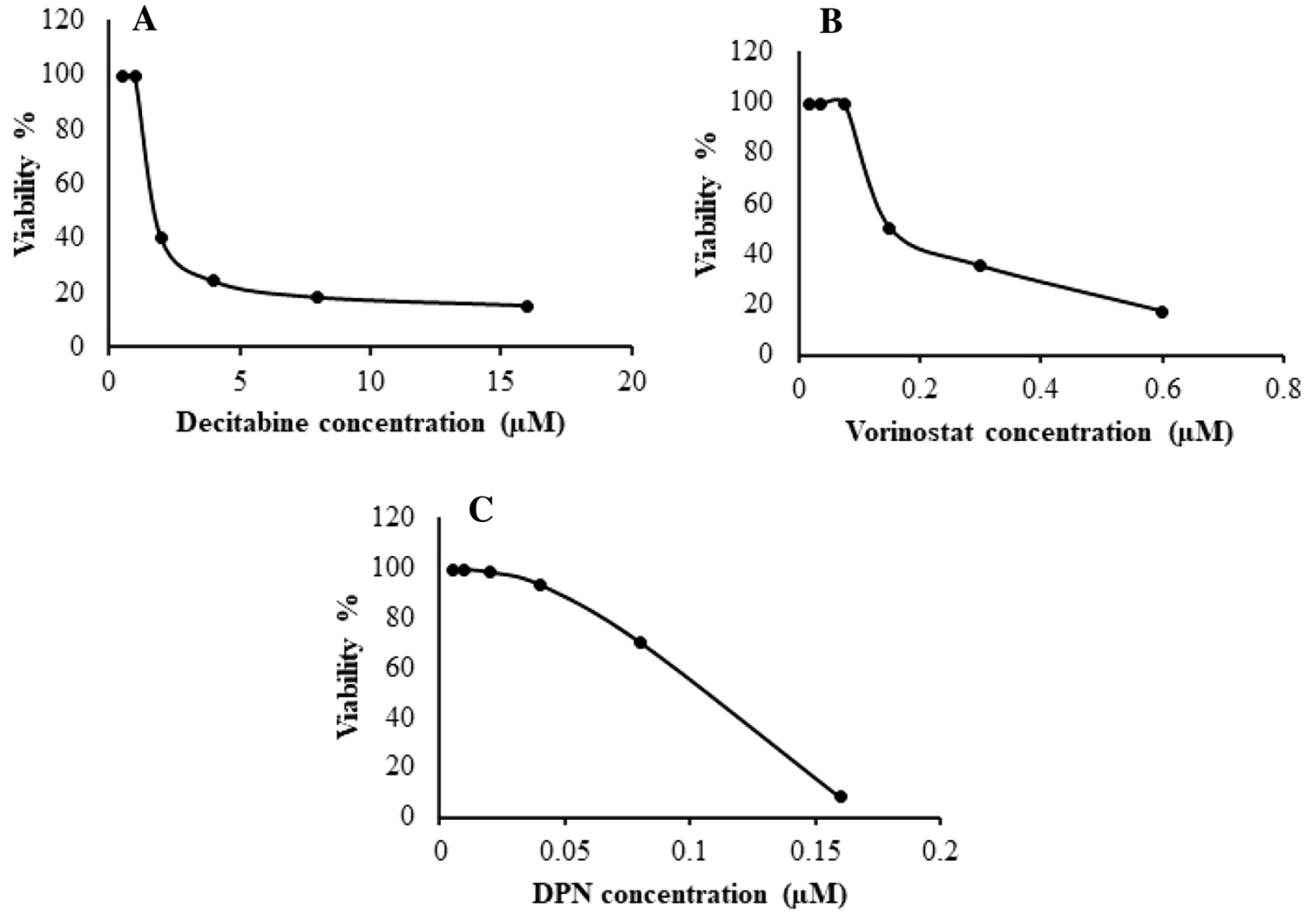
Sigmoidal curve for MTT assay showing IC_50_ values and the inhibition % of decitabine (**A**), vorinostat (**B**), and DPN (**C**) on MDA-MB-231 cells. Each data point represents an average of three independent experiments

Effects of decitabine, vorinostat, and DPN on MDA-MB-231 cells viability are shown in Fig. 1, respectively. Drugs showed concentration-dependent cytotoxic effects, where treating cells with decitabine concentrations (0.50, 1, 2, 4, 8, and 16 μM) inhibited the cell viability with an IC_50_ of 4 μM. Similarly, vorinostat treatment at different concentrations (0.0187, 0.0375, 0.075, 0.15, 0.30 and 0.60 μM) potently inhibited cell growth with an IC_50_ of 0.26 μM, and DPN at concentrations (0.005, 0.01, 0.02, 0.04, 0.08 and 0.16 μM) inhibited cell growth with an IC_50_ of 0.093 μM.

### Effects of decitabine, vorinostat, DPN and their combinations on ERα and ERβ genes expression in MDA-MB-231 cells

**Fig. 2:**
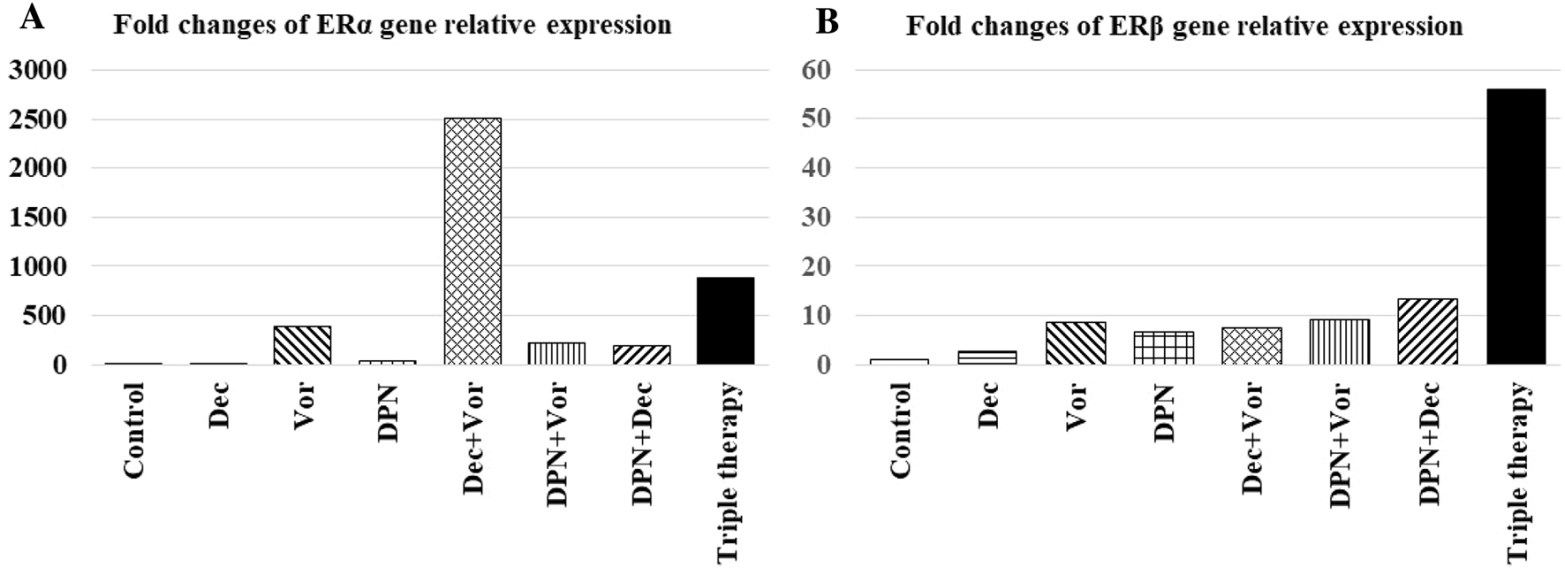
Fold changes of ERα (**A**) and ERβ (**B**) relative expression levels relative to the control group after treatment with decitabine (4 µM), vorinostat (0.26 µM), DPN (0.093 µM) and their combinations for 72 h in MDA-MB-231 cells

Figure 2 illustrates that incubation of MDA-MB-231 cells with decitabine, vorinostat, or DPN alone induced both ERα and ERβ mRNA expression at different levels in groups (II-IV) compared to the control group (I). Using a combination of decitabine and vorinostat as epigenetic drugs in the group (V) showed high induction of both ERα and ERβ genes expression with an obvious greater increase of ERα expression level by 220.9 and 6.4 folds compared to using decitabine or vorinostat alone, respectively. The addition of DPN (ERβ agonist) upregulated ERβ expression in all its combinations groups (VI-VIII) especially the triple combination therapy group (VIII), which showed the highest ERβ expression level of all treatment groups compared to the vehicle control group. On the other hand, DPN downregulated ERα expression in the triple therapy group (VIII) by 2.8-fold in comparison with its highest expression in the double epigenetic drugs combination (decitabine and vorinostat) in the group (V).

### ERβ expression and/or activation reduced cyclin D1 and IGF-1 protein levels when co-expressed with ERα in MDA-MB-231 cells

**Fig. 3:**
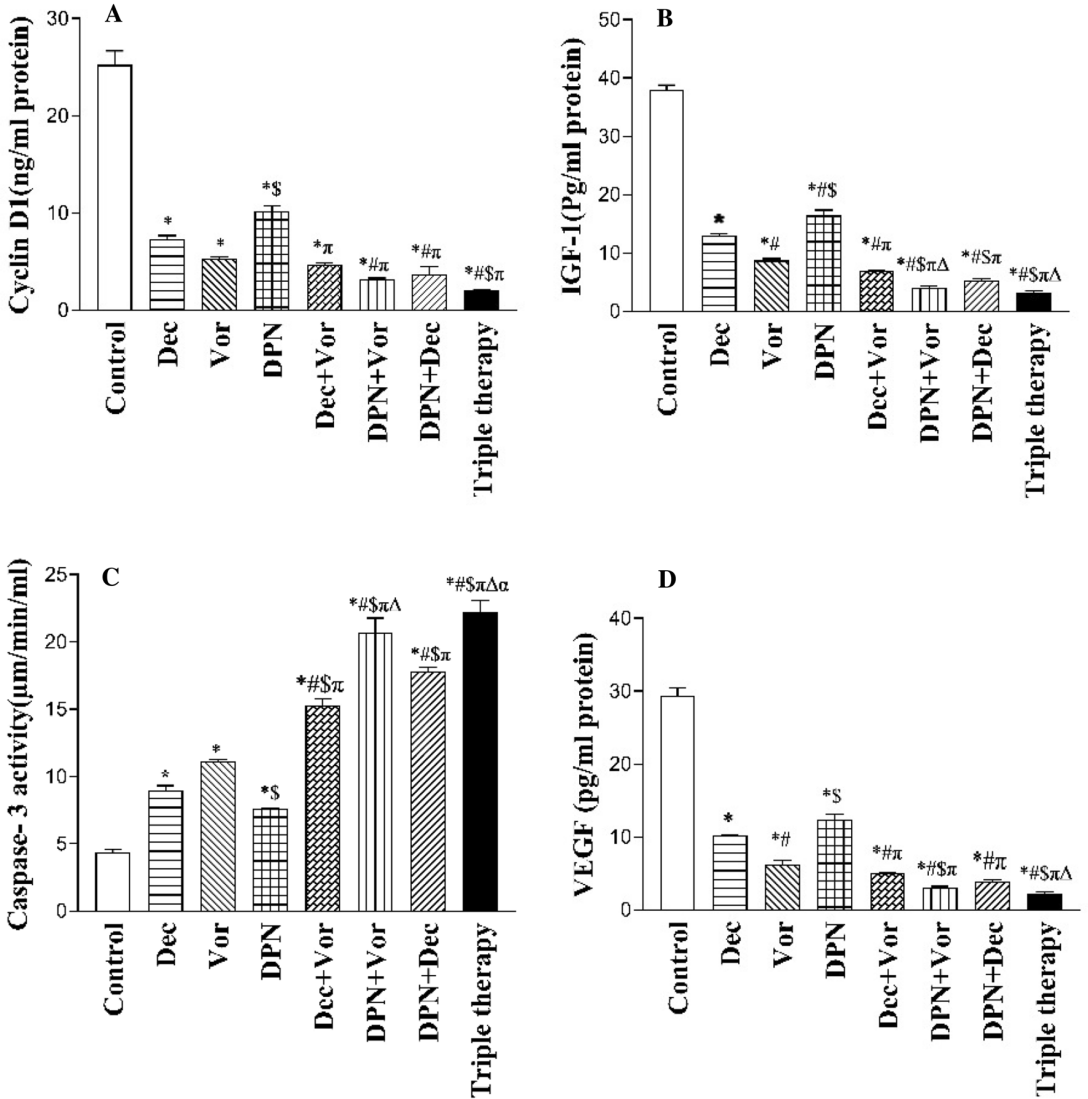
Effects of decitabine (4 µM), vorinostat (0.26 µM), DPN (0.093 µM) and their combinations treatments on the treated groups of MDA-MB-231 cells **A** cyclin D1, **B** IGF-1 **C** active caspase-3 activity, and **D** VEGF biomarkers. Data are presented as the mean ± SEM of three samples each performed in triplicate. Statistically significant differences between groups (*p* < 0.05) are designated as *significant vs*.* control, ^π^significant vs. DPN, ^#^significant vs. decitabine, ^$^significant vs. vorinostat, ^Δ^significant vs. decitabine & vorinostat

Figure 3A and B shows that decitabine, vorinostat, DPN, and their combinations affected the expression of both proliferation markers cyclin D1 and IGF-1 with different extents. Both cyclin D1 and IGF-1 proteins levels were significantly reduced (*p* < 0.001) in all treated cells groups (II–VIII) compared to their highest levels in the untreated control group (I).

It was noticed that cells treated with combination therapies groups (V–VIII) remarkably reduced both cyclin D1 and IGF-1 proteins levels more than using each drug alone, and the greatest reduction of cyclin D1 by 12.2-fold and of IGF-1 by11.6-fold compared to the control group was observed in cells that displayed the highest level of ERβ expression and activation, the triple therapy group (VIII). Hence, the data raised the idea that ERβ enhanced expression and/or activation can exert an antiproliferative effect when co-expressed with ERα in MDA-MB-231 cells.

### ERβ expression and/or activation stimulated apoptosis and enhanced caspase‑3 activity when co-expressed with ERα in MDA-MB-231 cells

To investigate the effect of decitabine, vorinostat, DPN and their combinations along with ERβ expression and/or activation on apoptosis, the caspase-3 activity was assessed as an apoptotic marker. Figure 3C shows that the caspase-3 activity significantly increased (*p* < 0.001) in all treated groups (II–VIII) in comparison with the lowest level observed in the control group (I). In addition, the caspase-3 activity in cells receiving combined treatments groups (V–VIII) was significantly higher (*p* < 0.001) than treated cells receiving single-agent treatments, specially DPN drug combinations.

Of interest, the highest caspase-3 activity was detected in cells treated with triple therapy, which exhibited the highest level of ERβ expression and activation, indicating the greatest apoptotic effect. These results suggested that ERβ expression and/or activation contributed to the induction of apoptosis in the presence of ERα expression in MDA-MB-231 cells.

### ERβ expression and/or activation influences angiogenesis by reducing VEGF protein levels when co-expressed with ERα in MDA-MB-231 cells

Figure 3D shows the effects of decitabine, vorinostat, DPN and their combinations on the angiogenesis marker VEGF protein level in MDA-MB-231 treated cells. VEGF protein levels in all treated groups (II–VIII) were significantly decreased (*p* < 0.001) compared to the highest level in the control group (I). Additionally, cells exposed to the combined treatments groups (V–VIII) showed a significant decrease over the single treatment action in groups (II–VI) compared to the control group. Interestingly, the lowest level of VEGF and so angiogenesis was observed in cells treated with the three-drug combination, which had the highest level of ERβ expression and activation.

## Discussion

Because of the lack of target receptors, TNBC patients do not benefit from hormonal or HER2-targeted therapies. TNBC tumors showed extensive promoter hypermethylation of epigenetic biomarker genes compared with other BC subtypes, it was reported that both ERs’ expression is epigenetically controlled [21].

However, there have been conflicting results concerning the function and clinical value of ERβ, especially in TNBC. Several studies suggested that ERβ positivity is of a favorable prognostic value for TNBC [7, 22], no prognostic value [23, 24], or worse prognosis and correlates with aggressive phenotypes [5, 25]. Potential reasons for these discrepancies can be since TNBC is a heterogeneous disease of different subtypes [5]; others suggested that due to the existence of at least five different ERβ receptor isoforms (ERβ1-5) in human BCs whose biological functions largely remain controversial [6]. Despite these discrepancies, several studies demonstrated the antitumor effect of ERβ activation using selective agonists in ERβ positive TNBCs, suggesting that endocrine therapy options targeting ERβ should be considered to treat patients with TNBC [11, 26]. Therefore, targeting ERβ after its re-expression using epigenetic drugs could be a promising therapeutic strategy of ERβ negative TNBCs treatment.

In the present study, we examined the effects of epigenetic drugs, decitabine as DNMTI and vorinostat as HDACI, and the ERβ agonist DPN on ERα and ERβ expressions in the MDA-MB-231 cells which is a well-characterized model of TNBC that do not endogenously express any form of the estrogen receptor neither ERβ nor ERα [6].

Our results showed that using each epigenetic drug alone (decitabine or vorinostat) caused re-expression of both ERα and ERβ mRNA at different levels with a higher effect of vorinostat than decitabine while using both decitabine and vorinostat as an epigenetic combination treatment showed a high re-expression effect of both receptors with a substantially greater increase of ERα expression than each drug alone as expected. That agreed with mounting studies which demonstrated the ability of HDACIs, such as vorinostat, to reactivate ERα expression at both transcriptional and protein levels in ER-negative BC cell lines like MDA-MB-231 and in different aggressive subtypes of TNBC through an epigenetic mechanism, and they sensitized cells to the anti-estrogen drug tamoxifen [12, 27]. Additionally, studies reported that HDACIs like trichostatin A induced re-expression of ERβ in BC cell lines, including TNBC cells, that was also observed in the ovary and prostate cancer cell lines [14, 28, 29]. In several studies, decitabine treatment was associated with an increased re-expression of ERβ in BC [30] and prostate cancer cells [14].

The observed greater effect of the epigenetic drugs combination compared to each drug alone corroborates with other studies which showed that the combination of DNMTIs and HDACIs demonstrated a synergistic effect on reactivation of silenced genes in cancer besides their beneficial anti-tumor effects [14, 29, 31], as shown in MDA-MB-231 and other TNBC cell lines through re-expression of ERs after application of this combination [12, 32].

In early clinical trials, decitabine did not show a major therapeutic effect when administered as monotherapy, however, preclinical and clinical studies of different cancer entities showed evidence of a synergistic effect of decitabine in combination with HDACIs to restore ERα expression and sensitize ER-negative BCs to hormone therapy or chemotherapy [33, 34]. Studies demonstrated that decitabine and trichostatin A co-administration potentiated the re-expression of ERβ in breast, ovary, and prostate cancer cell lines, and induced apoptosis, cell differentiation, and growth termination [14, 35]. Confirming that it was the best choice for our study to use this epigenetic combination (decitabine and vorinostat) together better than using each drug alone to re-express both ERs.

Additionally, DPN alone demonstrated an upregulation effect on ERβ expression. Similarly, several studies indicated that DPN induced ERβ expression in prostate cancer cells, which may be attributed to the receptor autoregulation caused by the presence of ERE sequences in the distant promoter region of the human ERβ gene [14, 36, 37]. This finding evoked hopes that ERβ agonists could be used clinically to upregulate ERβ expression in the early stages of cancer and thus prevent proliferation and progression [38].

The addition of DPN had a marked upregulation effect on ERβ expression in all of its combination groups (VI-VIII), especially the triple combination therapy group (VIII), which showed the highest ERβ expression level (56.04-fold) compared to the vehicle control group as well as to other treated groups. These data suggested an augmented effect between epigenetic treatment and receptor activation on ERβ expression in MDA-MB-231 cells. On the other hand, DPN as an ERβ agonist showed a negative effect and markedly decreased ERα re-expression levels, which appeared especially in the triple therapy group (VIII) compared to the highest expression level of ERα in (decitabine + vorinostat) epigenetic combination treatment group (V). That was consistent with several previous studies which indicated that ERβ downregulates ERα expression when co-expressed together via heterodimerization with ERα and increased ERα proteolytic degradation; as a result, ERβ can inhibit ERα activity and its proliferation effect [29, 39]. The antitumor effects of ERβ expression and activation were investigated and confirmed by measuring the other parameters.

Concerning the anti-proliferative effect of ERβ, the exact role of ERβ in BC is controversial; both proliferative and anti-proliferative ERβ roles have been described [40]. Our study aimed to examine the anti-proliferation effect of ERβ expression and activation when co-expressed with ERα, so we determined both cyclin D1 and IGF-1 protein levels in MDA-MB-231 cells as proliferation biomarkers.

Cyclin D1 is a cell cycle-related protein responsible for the transition from the (G1) phase to the (S) phase in the cell cycle. Its overexpression has been described in several human malignancies, including BC [14]. Alterations in IGF-1/IGF-1R signaling mediate stimulatory effects in malignant cells. High IGF-1R expression and elevated IGF-1 circulating levels have been correlated with the increased risk and progression of BC with poor prognosis through promoting cell proliferation, invasion, anti-apoptosis, and tumor angiogenesis [41, 42]. In particular, it had been shown that approximately 30–40% of TNBCs harbors amplification of the IGF-1R gene, which was linked to a short survival rate of these patients [43]. Additionally, high IGF-1 gene expression or IGF-1R (or both) levels were correlated with a worse clinical outcome in TNBC patients and triggered the growth potential, proliferation, and invasion of TNBC, including MDA-MB-231 contributing to the progression of more aggressive TNBC subtypes with poor survival [43–45].

Our results showed that re-expression of ERβ significantly reduced protein levels of both cyclin D1 and IGF-1 in all treated groups compared to the control group indicating the antiproliferative effect of ERβ expression. That was consistent with various studies that demonstrated the anti-proliferative effect of ERβ in MDA-MB-231 and MDA-MB-468 TNBC cell lines after ERβ exogenous expression and its activation by E2 or specific agonists such as DPN, which was able to notably inhibit TNBC cell growth, arrest cell cycle at the G1 phase, block cell colony formation, inhibit cell invasiveness and reduce tumor size in mice xenografts [11, 46]. A recent study confirmed that overexpression of ERβ using adenoviral infection as a means to elevate ERβ levels was found to suppress the proliferation, migration, and invasion of the MDA-MB-231 cells and in other TNBC subtypes [47] consistent with other studies which found that ERβ had a tumor-suppressive effect [11, 48].

In contrast to our results, some studies reported that ERβ overexpression increased the rate of cell proliferation and progression in some TNBC subtypes; in addition, ERβ positivity in TNBC was correlated with higher expression of the proliferation marker Ki- 67 [5]. In another study, targeting ERβ with DPN in T47D cells (ERα- positive/ERβ1-positive) had a little to no effect on the proliferation rates [6, 49].

Interestingly, our results indicated that the re-expression of ERβ and ERα together in high levels using the epigenetic drugs combination group (V) displayed more reduction of cyclin D1 and IGF-1 protein levels than decitabine, vorinostat, or DPN alone (groups II, III, and IV, respectively). This antiproliferative effect was markedly enhanced through ERβ activation using its agonist DPN combined with the epigenetic drugs, especially in the triple therapy group (VIII), which displayed the highest ERβ expression and so the highest antiproliferation effect. Consequently, these results proved that increasing ERβ expression level using the epigenetic drugs combined with its activation had a crucial role in obtaining a stronger ERβ antiproliferative effect and better than using ERβ agonist alone.

It was important to note that co-expression of both ERs together had a substantial effect on ERβ actions. In contrast to the confused roles of ERβ in TNBC when expressed alone, data suggested that the biological effects of ERβ are critically correlated with the presence of ERα [5, 6]. ERβ appeared to oppose ERα actions on cell proliferation by modulating the expression of many ERα regulated genes [50]. When both ERs co-expressed in cells, ERβ can antagonize ERα-dependent transcription and inhibit ERα proliferative role via alteration of key transcription factors recruitment, heterodimerization and increase ERα proteolytic degradation [29, 39]. In this context, studies showed that ERα- positive/ERβ1-positive tumors typically had reduced expression of Ki67 relative to ERα-positive/ERβ1-negative tumors confirming the tumor suppressor role of ERβ in the ERα-positive BC cell lines; additionally, ERβ expression diminished the pro-proliferative effects of ERα and exerted its oncosuppressive role targeting cell division [51, 52].

Gene expression analysis reported that gene expression of cyclin D1 is regulated by estrogen via AP-1 site, which is stimulated by ERα, while is inhibited by ERβ, suggesting that ERβ may modulate the proliferative effects of ERα by blocking its action on the cyclin D1 gene [53]. In tune, a study of a human cervical cancer cell line (Hela cells) had previously demonstrated that E2-activated ERβ acted as a negative regulator of cyclin D1 gene transcription and effectively abrogated the ER-α-mediated activation of cyclin D1 expression when both ER subtypes are co-expressed [54].

These reports collectively were consistent with our finding that targeting the highest level of ERβ with DPN, after its co-expression with ERα using the epigenetic drugs in the triple therapy group, was a crucial step that resulted in obtaining the greatest antiproliferative effect among all treated groups.

Concerning the effect of ERβ re-expression and activation in the presence of ERα on apoptosis, treatment with either decitabine, vorinostat, or DPN alone significantly enhanced caspase-3 activity which is a member of the proteases family that mediate cell death and is one of the critical enzymes of the apoptosis process [55]. This observation supported the belief that ERβ exerts apoptotic effects on various malignant cells [56, 57]. The majority of data from the research on clinical samples and cell lines suggested that ERβ has antiproliferative, tumor-suppressive functions and induces apoptosis in ERα low or negative BC cell lines [29, 58]. Exogenous expression of ERβ was reported to exert apoptotic effects in prostate carcinoma cells [59].

This work showed that cells exposed to combination therapies in groups (V–VIII) exhibited significantly higher levels of caspase-3 activity compared to the single agents applied alone and to the vehicle control group. In tune, studies demonstrated that co-treatment of prostate cancer cells with (decitabine + trichostatin A) was associated with a significant increase in apoptotic activity compared with the single agents alone [14], an effect that seemed to be related to increased ERβ expression. Remarkably, in the present study, the highest level of ERβ expression and activation (in the presence of ERα) in the three-drug combination group was accompanied by the highest level of caspase-3 activity and so apoptosis. The same results were observed after induction of ERβ expression in a prostate cancer cell line in a study that followed the same idea of our work about using a triple therapy of epigenetic drugs and ERβ agonist (decitabine + trichostatin A + DPN) [14]. This observation suggested that triple therapy has the greatest tendency to induce apoptosis via activation of the highest level re-expressed ERβ in the presence of ERα in MDA-MB-231 cells.

Angiogenesis was also estimated by measuring VEGF, which is a key regulator of angiogenesis and can stimulate endothelial cell proliferation to form new blood vessels that support tumor growth and increase the risk of tumor invasion, metastasis, and patient mortality [60]. VEGF overexpression has been described in solid malignancies, including BC [61, 62]. Previous studies have shown that TNBC possesses high microvessel density and VEGF amplification than non-TNBC [62, 63]. Herein, individual administration of decitabine, vorinostat, or DPN alone caused a significant reduction in VEGF protein level as compared to the control group, but cells exposed to the combined treatments showed a significant decrease over the single-agent action. That was attributed to the increased levels of ERβ expression and activation in the presence of ERα, in addition to the antitumor effect of the epigenetic drugs.

That was in agreement with data that revealed that the specific HDACI, entinostat, attenuated tumor progression and metastasis in TNBC through downregulation of VEGF expression and enhancing the re-expression of anti-angiogenic and tumor suppressor genes epigenetically [62], supporting the antiangiogenic effect of our epigenetic drugs.

In addition, estrogens had been implicated in controlling VEGF expression in target tissues and corresponding tumors through ERα and ERβ [64]. Liu et al*.* showed that liquiritigenin, an ERβ agonist, reduced tumor growth of HeLa cells in nude mice via inhibition of VEGF expression and so angiogenesis [65]. Similarly, Motawi et al*.* have demonstrated that re-expression of ERβ followed by activation using DPN treatment attenuated VEGF protein level in the PC-3 prostate cancer cells [14]. Interestingly, in our study, the lowest levels of VEGF and so angiogenesis was observed in cells treated with the three-drug combination, which had the highest levels of ERβ re-expression, supporting that ERβ re-expression and activation are responsible, at least in part, for the downregulation of VEGF protein expression that would eventually repress angiogenesis in MDA-MB-231 cells.

## Conclusion

Taken all together, according to the aforementioned evidence, the combinatorial therapy of decitabine, vorinostat, and DPN implied retaining the anti-tumor effect of ERβ as a result of induced ERβ overexpression and activation in the presence of ERα in MDA-MB231 TNBC cells. That may hold promises for those patients with extremely poor outcomes and for which no form of targeted cancer therapy is currently available. Therefore, we recommend further preclinical and clinical studies on different subtypes of TNBC cells to verify the validity of such a promising triple combination. The same notion should be further elevated in other hormone-dependent cancers like prostate, endometrial, and cervical cancer.

## Abbreviations

BC: Breast cancer
DMEM: Dulbecco's modified eagle's medium
DMSO: Dimethyl sulfoxide
DNMTI: DNA methyltransferase inhibitor
DNMTs: DNA methyltransferases
DPN: (2,3-Bis (4-hydroxyphenyl) propionitrile)
Erα: Estrogen receptor alpha
Erβ: Estrogen receptor beta
FBS: Fetal bovine serum
GAPDH: Glyceraldehyde-3-phosphate dehydrogenase
HDACI: Vorinostat as histone deacetylase inhibitor
HDACs: Histone deacetylases
HER2: Human epidermal growth factor receptor 2
IC50: Median inhibitory concentration
IGF-1: Insulin-like growth factor 1
MTT: 3-(4, 5-Dimethyl thiazolyl-2)-2, 5-diphenyltetrazolium bromide
PBS: Phosphate buffer saline
PR: Progesterone receptor
qRT‑PCR: Quantitative real‑time polymerase chain reaction
TNBC: Triple-negative breast cancer
VEGF: Vascular endothelial growth factor
